# ALT cancer cells are specifically sensitive to lysine acetyl transferase inhibition

**DOI:** 10.18632/oncotarget.26616

**Published:** 2019-01-22

**Authors:** Dalal Bakhos-Douaihy, Chantal Desmaze, Maya Jeitany, Laurent R. Gauthier, Denis Biard, Marie-Pierre Junier, Hervé Chneiweiss, François D. Boussin

**Affiliations:** ^1^ Laboratoire de Radiopathologie, CEA, Institut de Radiobiologie Cellulaire et Moléculaire, Fontenay-aux-Roses, France; ^2^ INSERM U1276, Fontenay-aux-Roses, France; ^3^ Université Paris-Diderot, U1276, Fontenay-aux-Roses, France; ^4^ Université Paris-Sud, U1276, Fontenay-aux-Roses, France; ^5^ CEA, Institut de Biologie François Jacob, SEPIA, Team Cellular Engineering and Human Syndromes, Université Paris-Saclay, F-92265 Fontenay-aux-Roses, France; ^6^ Neuroscience Paris Seine-IBPS, CNRS UMR8246, Inserm U1130, Sorbonne Université, Paris, France

**Keywords:** alternative mechanism of telomere maintenance, PCAF, GCN5, ionizing radiation

## Abstract

Some cancer cells elongate their telomeres through the ALT (alternative lengthening of telomeres) pathway, which is based on homologous recombination for the addition of telomere repeats without telomerase activity. *General control non-derepressible 5 (GCN5) and P300/CBP-associated factor (PCAF),* two homologous lysine acetyltransferases, exert opposite effects on the ALT pathway, inhibiting or favoring it respectively. Here we show that ALT cells are particularly sensitive to the inhibition of acetyltransferases activities using Anacardic Acid (AA). AA treatment recapitulates the effect of PCAF knockdown on several ALT features, suggesting that AA decreased the ALT mechanism through the inhibition of lysine transferase activity of PCAF, but not that of GCN5. Furthermore, AA specifically sensitizes human ALT cells to radiation as compared to telomerase-positive cells suggesting that the inhibition of lysine acetyltransferases activity may be used to increase the radiotherapy efficiency against ALT cancers.

## INTRODUCTION

Some cancer cells counteract the telomere attrition occurring through cell division not by activating telomerase but through the ALT (alternative lengthening of telomeres) pathway. ALT pathway has been observed in various types of human tumors such as sarcomas and gliomas [1, 2]. ALT is based on homologous recombination for the addition of telomere repeats [3]. Telomeres of ALT cells are highly heterogeneous in length, ranging from undetectable to extremely long [4]. ALT cells contain specialized promyelocytic leukemia (PML) nuclear bodies termed ALT-associated PML bodies (APBs), which are thought to be the main sites of telomere elongation in ALT cells. APBs contain usual PML nuclear bodies components like PML and Sp100 along with telomeric DNA, telomere binding proteins, and a mixture of DNA replication, recombination and repair factors [5, 6]. ALT cells are also characterized by the presence of abundant linear and circular extrachromosomal telomere repeats (ECTR) [7, 8]. Finally, the ALT telomeres are submitted to a high level of post-replicative exchanges known as telomere-sister chromatid exchanges (T-SCEs) [9].

The histone acetyltransferases (HATs) General control non-derepressible 5 (GCN5) and P300/CBP-associated factor (PCAF) share ∼73% amino acid sequence identity and play a major role in the regulation of transcription. Several studies have shown that histones are not the only target substrate for these two enzymes. These two proteins are mutually exclusive subunits of large complexes such as SAGA-like or Ada-Two-A-containing (ATAC)-like [10, 11]. These complexes are involved in the regulation of distinct substrates and they have multiple biological roles such as participating in the DNA damage response and telomere maintenance mechanism [12, 13]. Indeed, in telomerase-positive cells, GCN5 has been shown to be a key player in the homeostasis of chromosome end by preventing signaling associated with telomere DNA damage and thereby protecting telomeres from fusions [12]. We have recently shown that GCN5 knockdown increased T-SCE, and telomere instability in ALT cells, whereas PCAF knockdown had opposite effects decreasing T-SCE, APBs formation and telomere instability [13]. However, the impact of the PCAF and GCN5 lysine acetyl transferase activities on the ALT mechanism remains unknown.

In this report, we used anacardic acid (AA), a pan-inhibitor of histone acetyl transferases (HATs) activities [14, 15] to investigate the role of PCAF and GCN5 enzymatic activity in the regulation of ALT. AA occurs naturally in cashew nuts (the most accessible natural dietary source), mangos, and cashew apples. Diverse biological activities for the AAs have been described, including antitumor activity against various cancers such as prostate cancer [16], breast cancer cells [17], or pituitary adenoma cells [18]. Our results show that AA inhibits ALT, eventually affecting ALT cell growth and viability. We demonstrate that PCAF but not GCN5 acetyl transferase activity, is involved in this process. Furthermore, we show that AA specifically radio-sensitizes ALT cells, as compared to telomerase-positive cell lines.

## RESULTS

### AA decreased cell growth and viability of the ALT cell lines TG20 and SAOS2

**Figure 1:**
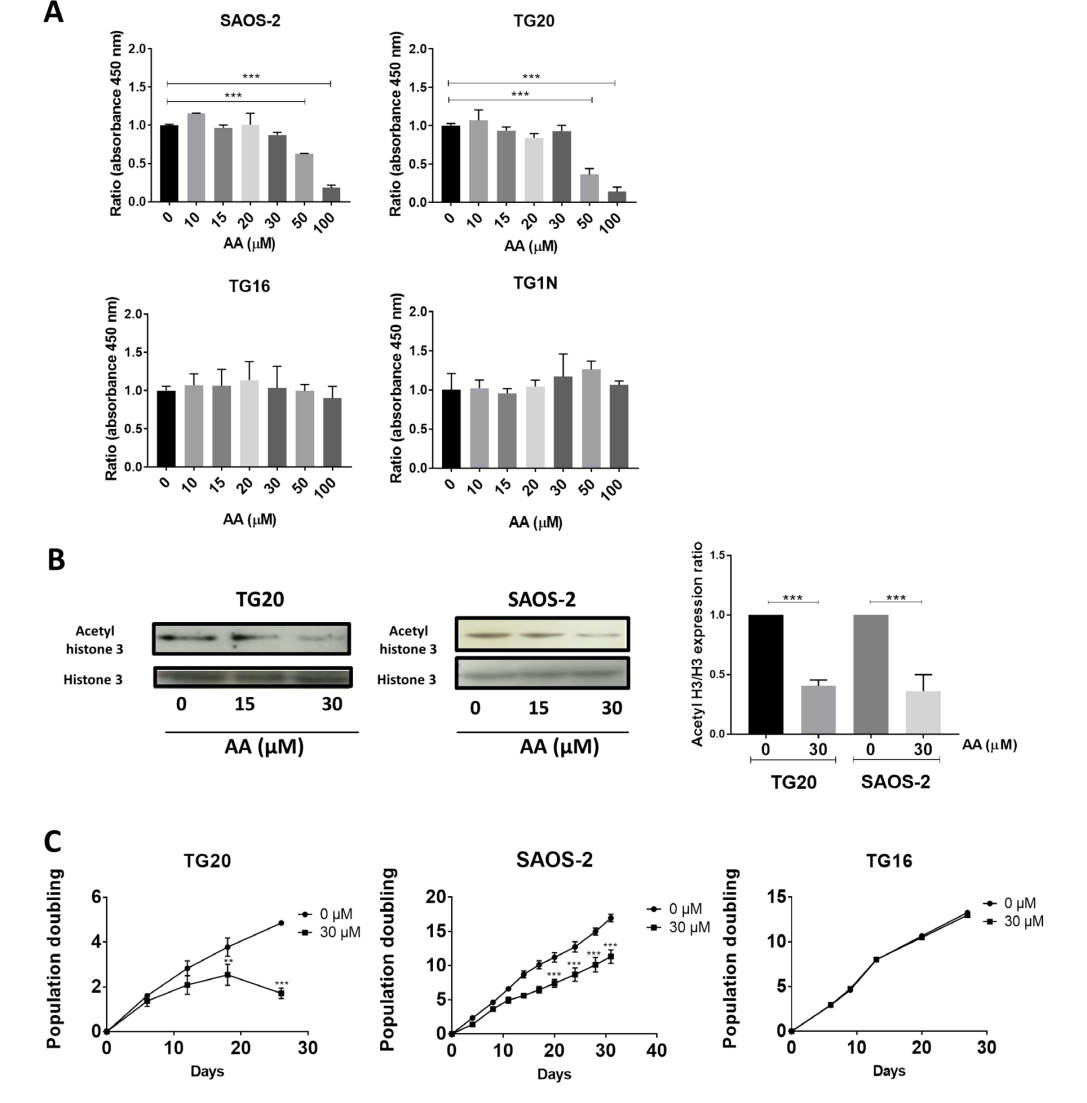
Comparative short and long-term effects of AA on cell growth of ALT and telomerase positive cells (**A**) The short-term cytotoxicity of AA in a broad concentration range in both ALT and telomerase positive cells was evaluated. The percentages of viable SAOS-2, TG20, TG16, and TG1N cells were determined using the cell proliferation reagent WST-1 72 h after AA treatment (10 to 100 μM). Cultures treated with the vehicle (0.1% DMSO) were used as controls. To calculate cell viability, the value of the signal from the treated culture wells were expressed as a ratio of that of the control wells. Results are shown as mean ± SD from triplicates. The experiments were repeated 3 times. ^***^p < 0.001, one way Anova. (**B**) The effect of AA on acetylated histone 3 in ALT cells was analyzed. Western blot analysis of acetylated forms of histone H3 and total content of histone H3 was performed in both ALT (SAOS-2 and TG20) cell extracts 72 h after 15 and 30 µM AA treatments (left). The untreated controls contained DMSO. The quantitative data (right) are shown as relative intensity of acetylated histone band in arbitrary units that was adjusted for total histone 3 intensity and normalized to those of the control untreated. Data are expressed as the means ± SD of two independent experiments for each cell line. ^***^P < 0.001 compared with vehicle-treated cells, Tukey-Kramer one way Anova. (**C**) Population doubling (PD) curves of TG20, SAOS-2 and TG16 cell lines. Cells were continuously cultivated in the presence of AA (30 µM) for 30 days, and the cell growth was monitored. Cells treated with DMSO were used as a control. The x-axis indicates the number of incubation days, and the y-axis indicates the number of population doublings. Black circles: vehicle-treated cells. Black squares: AA-treated cells. Viable cells were counted weekly by trypan blue staining using a Malassez cell. Population doublings were calculated by the formula log [(number of cells harvested)/(number of cells seeded)]/log2. Each curve depicts the averaged results (+SD) from two different experiments. ^**^p < 0.01, ^***^p < 0.001, 2-way ANOVA test.

We first investigated cell viability after 72 h of treatment with increasing doses of AA by using the WST-1 assay. As shown in Figure 1A, concentrations up to 30 µM of AA had no significant effect on the viability of any of the cell lines tested. Interestingly, 100 µM AA had no effect on the telomerase-positive GSCs (TG1N and TG16), but dramatically decreased the viability of ALT cells, SAOS-2 and TG20. Consistently, detection of cleaved-caspase 3-positive cells showed the induction of apoptosis in ALT cells treated with 100 μM AA, but not with 30 μM AA, whereas no increase in apoptosis was detected in telomerase-positive GSCs treated with any concentrations of AA (Supplementary Figure 1).

We then analyzed the effects of AA on lysine acetylation in two telomerase-positive cell lines (TG1N and TG16 [19]) and two ALT cell lines (TG20 [19, 20], and SAOS2 (HTB85, ATCC). To this end, we measured the levels of lysine acetylation of histone H3 known to be the preferred substrate of both PCAF and GCN5 acetyltransferase activities [21, 22]. Western blotting using an anti-acetyl-Histone H3 antibody showed that 30 µM AA significantly decreased by 55 to 78% Histone H3 acetylation after 72 h of treatment in both ALT (SAOS-2 and TG20) (Figure 1B) and telomerase-positive (TG16 and TG1N) (Supplementary Figure 2) cells.

We next determined the effects of long-term treatments with 30 µM AA on cell growth. As shown in Figure 1C, AA had no effect on population doublings in cultures of the telomerase-positive GSCs TG16. On the opposite, AA significantly decreased the growth of the ALT cell lines (SAOS-2 and TG20), with TG20 being the most sensitive.

Altogether, these data suggest that ALT cell lines are specifically sensitive to Lysine acetyl transferases inhibition by AA as compared to telomerase-positive cell lines.

### AA downregulates ALT

**Figure 2:**
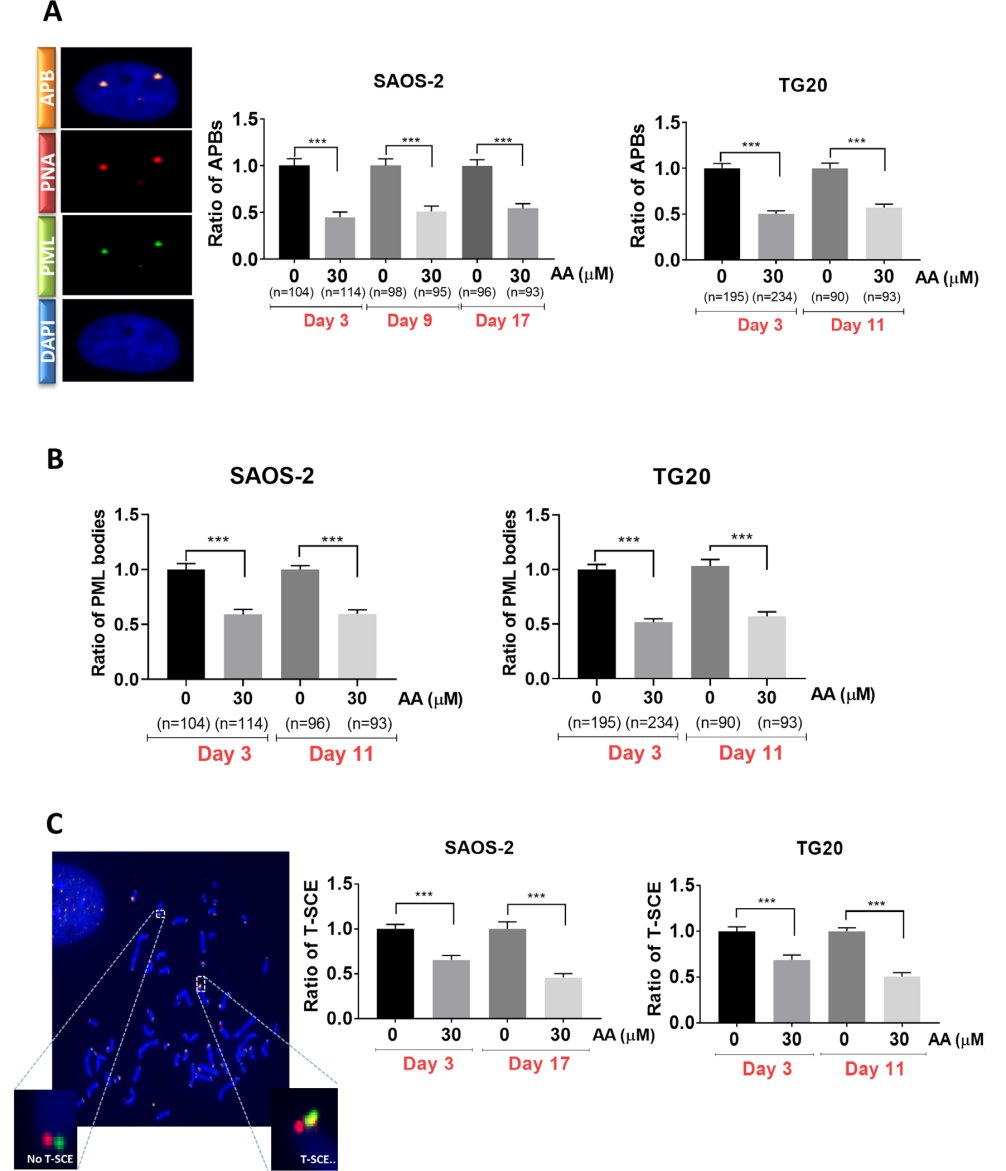
Long term AA treatment is associated with suppression of ALT activity (**A**) Representative images of APB (left) in SAOS-2, captured with confocal microscopy. One APB is detected by double immunostaining of PML bodies (green) and telomere (Cy-3-labeled (CCCTAA)3 PNA probe) (red). Cells were treated with 30 µM AA for 30 days. Cells treated with DMSO were used as a control. APBs were counted in SAOS-2 (at day 3, day 9 and day 17) (middle) and TG20 (at day 3 and day 11) (right). “n” indicates the number of counted cells. The values represent the ratio of number of APBs per cell (+SEM) relative to untreated control for each cell line and day of treatment. ^***^p < 0.001, Student’s *t*-test. (**B**) PML bodies were scored in SAOS-2 (at day 3 and day 17) (left) and TG20 (at day 3 and day 11) (right) treated or not with AA. “n” indicates the number of counted cells. The values represent the ratio of number of PML per cell (+SEM) relative to untreated for each cell line and day of treatment. (^***^p < 0.001 as determined by Student’s *t*-test). (**C**) Illustrative telomeric exchange events (left) at TG20 telomeres using the CO-FISH technique. The differentially-labeled sister chromatids using two parental strand specific probes: an FITC-labelled (TTAGGG)3 PNA probe (green) and a Cy-3-labeled (CCCTAA)3 PNA probe (red) were visualized using fluorescence microscopy. In the case of exchange, the two probes will hybridize on the same strand and T-SCE (yellow) can thus be quantified. The T-SCE events were scored at early and late days of AA treatment in TG20 (right) and SAOS-2 (middle). The values are the ratio of T-SCE events (+SEM) relative to untreated control for each condition. Between 2000 and 3000 chromosome extremities were analyzed (^***^p < 0.001, as reported by Student’s *t*-test).

We thus sought to determine whether the effects of AA on cell growth and viability were associated with interferences with the ALT pathway. To this end we scored the number of APBs in cells treated with AA for different time periods. APBs are PML bodies in which telomeres are elongated and are thus specific of ALT cells [23]. As shown in Figure 2A, the mean numbers of PML bodies co-localizing with telomeres, were constantly decreased by nearly 50% in both TG20 and SAOS2 cells treated with 30 µM AA as compared to untreated controls.

Interestingly, immunofluorescence analysis showed that the number of PML bodies was reduced by 50% after 72 h of AA treatment in both ALT cell lines as compared to non-treated cells (Figure 2B). By contrast, AA had no effect on the number of PML bodies in telomerase-positive TG16 cells (Supplementary Figure 3). Our results reveal thus that the decrease in APBs caused by AA was associated with a reduction in the amount of PML bodies specific to ALT cells.

We next investigated whether AA interfered with T-SCE, another hallmark of ALT cells, by performing chromosome orientation fluorescence *in situ* hybridization (Co–FISH) on metaphase chromosomes as previously described [24, 25]. As shown in Figure 2C, the frequency of T-SCE was significantly decreased in SAOS-2 and TG20 cells treated with 30 µM of AA for 3 (30% reduction) or 10 to 17 days (50% reduction).

The decrease in cell growth and viability induced by inhibition of lysine acetyl transferases in AA-treated SAOS2 and TG20 cells is thus clearly associated to a down regulation of the ALT mechanism.

### AA decreased ALT through the inhibition of the lysine acetyl transferase activity of PCAF, but not that of GCN5

In order to determine whether the lysine acetyl transferase activities of PCAF and GCN5 are involved in the regulation of ALT, we analyzed the effects of AA on APB formation and T-SCEs in TG20 and SAOS-2 cells knocked down for either PCAF or GCN5 using specific siRNAs (siPCAF or siGCN5 respectively, Supplementary Figure 4A) as previously described [13].

**Figure 3:**
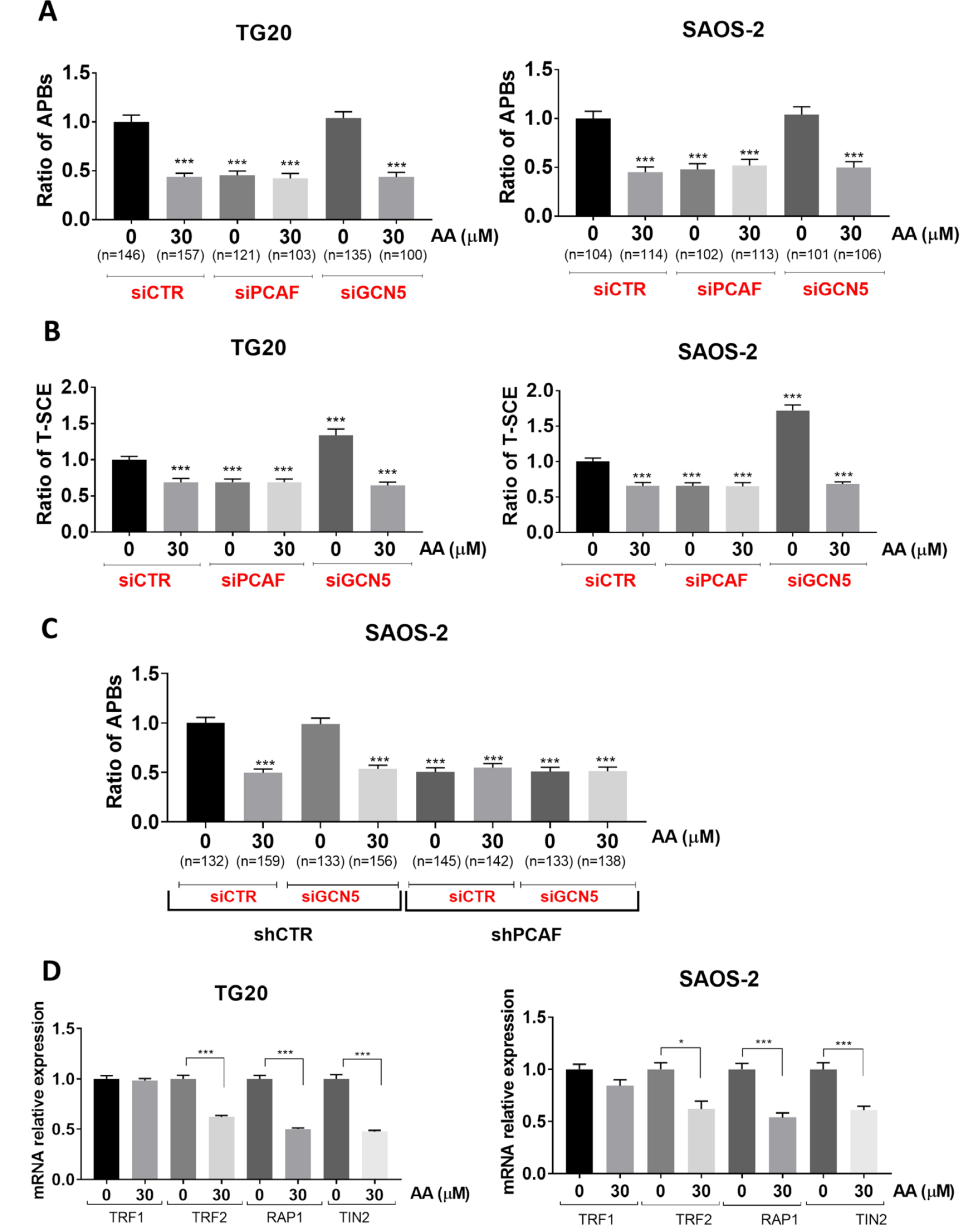
Specific implication of the lysine acetyl transferase activity of PCAF in the regulation of the ALT mechanism (**A**) APBs were scored at 72 h after transfection with siCTR, siPCAF and siGCN5 in TG20 (left) and SAOS-2 (right) treated or not with AA. “n” indicates the number of counted cells. The values represent the ratio of number of APBs per cell (+SEM) relative to untreated siCTR for each cell line. (^***^p < 0.001 as determined by Student’s *t*-test). (**B**) Quantification of TSC-E 72 h after PCAF or GCN5 down-regulation in TG20 (left) and SAOS-2 (right) cells treated or not with AA. The values are the ratio of T-SCE events (+SEM) relative to untreated siCTR for each cell line. Between 2000 and 3000 chromosome extremities were analyzed (^***^p < 0.001, as reported by Student’s *t*-test). (**C**) APBs were scored after transfection with siCTR or siGCN5 in SAOS-2 shCTR (left) and SAOS-2 shPCAF (right) treated or not with AA. “*n*” indicates the number of counted cells. The values represent the ratio of number of APBs per cell (+SEM) relative to untreated siCTR for each cell line. (^***^p < 0.001 as determined by Student’s *t*-test). (**D**) mRNA expression levels of TRF1, TRF2, TIN2 and RAP1 as determined by RT-PCR in TG20 (A) and SAOS-2 (B) cells treated with AA for 72 h relative to their expression in non-treated cells. The error bars are + SEM of at least two duplicates (^*^p < 0.05, ^***^p < 0.001 as determined by the *t*-test).

As shown in Figure 3, similar data were obtained with TG20 and SAOS-2 cells. Consistently with our previous report [13], PCAF knockdown significantly decreased the number of APBs, as compared to that found in cells transfected with control siRNAs (SiCtrl), whereas GCN5 knockdown had no effect (Figure 3A). Interestingly, treatment with 30 µM AA decreased APBs formation in cells transfected with either siCtrl, siPCAF or siGCN5 at a level similar to that found in vehicle-treated PCAF knockdown cells.

As previously demonstrated [13], PCAF knockdown decreased T-SCE as compared to siCTR transfected cells (Figure 3B). T-SCE were also decreased to similar extents in AA-treated cells transfected with either siCtrl-treated or siPCAF (Figure 3B). AA mimicked thus the inhibitory effects of PCAF knockdown on the formation of APBs and T-SCEs, two hallmarks of ALT. No additive or synergistic effects were observed when treating cells with both siPCAF and AA. As previously reported [13], GCN5 knockdown increased significantly the numbers of T-SCE as compared to siCtrl in both cell lines, but addition of 30 µM AA, decreased T-SCE at the same levels as the ones observed with siPCAF or with AA or combination of both (Figure 3B).

To give further evidence for a possible direct role of lysine acetyl transferase activity of PCAF on ALT, we performed the double inactivation of PCAF and GCN5. To this end, we have generated SAOS-2 cells stably expressing shRNA targeted against PCAF (named thereafter SAOS-2 shPCAF). We showed that mRNA and protein expression levels of PCAF were stably decreased in SAOS-2 shPCAF cells compared to control cells transfected with an inactive shRNA (named thereafter SAOS-2 shCTR) (Supplementary Figure 4B). We thus scored the number of APBs in cells knockdown for PCAF or GCN5 alone or both PCAF and GCN5 treated or not with AA. We found that the number of APBs was decreased in SAOS-2 shPCAF cells, as compared to SAOS-2 shCTR. Moreover, siGCN5 had no effect on APB formation in both SAOS-2 shCTR cells and SAOS-2 shPCAF cells. Finally, 30 µM AA decreased APB formation at similar levels to that of SAOS-2 shPCAF and has no effect on SAOS-2 shPCAF, whatever the GCN5 status (Figure 3C). Therefore, these experiments confirm the importance of PCAF lysine acetyl transferase activity and the lack of involvement of GCN5 lysine acetyl transferase activity in APB formation.

Since PCAF is known to be involved in transcriptional regulation of some genes *via* histone acetylation, we tested the effects of AA on the expression of genes involved in APB formation. We have thus quantified mRNA levels of TRF1, TRF2, TIN2 and RAP1, four proteins of the shelterin complex required for the assembly of APBs [26] by RT-qPCR on SAOS-2 and TG20 cells treated for 72h with AA. As shown in Figure 3D, TRF2, TIN2 and RAP1 mRNAs decreased by 36 to 50% in TG20 and SAOS_2 treated cells as compared to untreated cells. By contrasts, AA induced no obvious alterations of TRF1 mRNA levels (*p* > 0.05). These results suggest that the molecular mechanism of action of AA on ALT phenotype depends on transcriptional modulation of telomeric genes involved in APB formation and hence in ALT telomere lengthening.

Altogether our data suggest that AA decreased the ALT mechanism through the inhibition of lysine acetyl transferase activity of PCAF, but not that of GCN5.

### AA sensitizes ALT cells to irradiation

**Figure 4:**
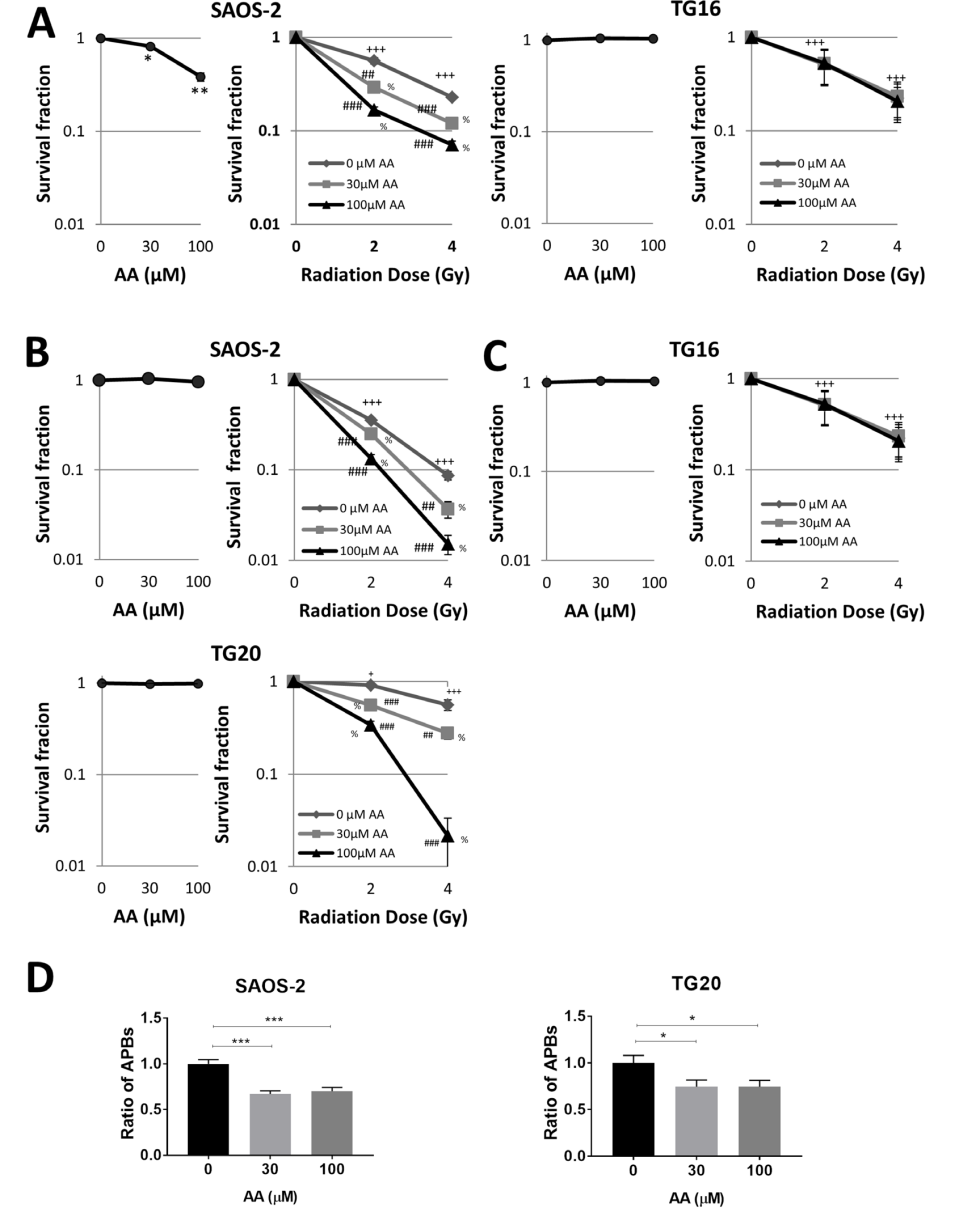
AA sensitizes ALT cells to irradiation (**A**–**C**) The colony formation assay for evaluation of the growth of ALT and telomerase positive cells upon AA treatment combined or not with irradiation was observed. (A) SAOS-2 ALT cells (left) and TG16 telomerase positive cells (right) were incubated with DMSO or AA for 72 h (left curve) and then irradiated at 0, 2 or 4 Gy (0.81 Gy/min) (right curve). (**B**–**C**) Cells were incubated with DMSO or AA for 24 h (left curve) and then irradiated at the indicated dose (right curve). Cells were then switched to fresh media and allowed to grow for 14 days. The colonies that contained n >10 cells were then counted to determine the colony formation ratio (number of colonies/number of seeded cells) of each group. The survival fraction was calculated as the colony formation ratio of each treated group divided by the colony formation ratio of the untreated control group. Data are expressed as mean ± SEM from three experiments (^***^P < 0.01,^**^P < 0.01 versus untreated controls, ^+^*P <* 0.05, ^+++^*P <* 0.001 for each radiation dose versus non-irradiated controls. ^##^*P <* 0.01, ^###^*P <* 0.001 for each radiation dose in combination with AA versus each radiation dose alone. % *P <* 0.001 for each AA concentration in combination with the same radiation dose versus control untreated, as determined by *t*-test). (**D**) SAOS-2 and TG20 ALT cells (left and right respectively) were pretreated with AA for 24 h. APBs were scored after the treatment. The values represent the ratio of number of APBs per cell (+SEM) relative to untreated, between 150 and 250 cells were counted in each condition. (^***^P < 0.01,^**^P < 0.01 and ^*^P < 0.05, as determined by Student’s *t*-test).

AA has been previously shown to sensitize various cancer cell lines to ionizing radiation, including the ALT cell line U20S [27, 28]. In order to investigate further how AA interferes with the cell response to radiation, SAOS-2 and TG16 cells were treated either with 30 µM or 100 µM AA for 3 days prior to be irradiated at 2 or 4 Gy. A clonogenicity assay was then performed in fresh medium without AA. As shown on Figure 4A, the colony formation assay confirmed the dose-dependent effect of AA on SAOS-2 cell viability and the lack of AA effect on the telomerase-positive TG16 cells. By contrast, irradiation similarly impaired the clonogenicity of both cell lines in a dose-dependent manner. Combining the two treatments revealed additive effects on ALT cells, whereas AA did not radio-sensitize the telomerase-positive cell lines tested. Since in these experiments radiosensitization could have been masked by the effects of AA on ALT cell proliferation and viability at the time of irradiation, we performed the irradiation and the clonogenicity assay after 24 h of treatment with AA. Results showed that the treatment with AA alone did not impair the clonogenicity of TG20 and SAOS-2 (Figure 4B), but induced a significant decrease in the number of APBs in both cell lines (Figure 4D). No radiosensitization of the telomerase-positive GSC lines TG16 was observed (Figure 4C). However, 24 h pretreatment with AA clearly radiosensitized the two ALT cell lines, TG20 and SAOS-2 (Figure 4B), reducing their clonogenicity in a dose-dependent manner. Altogether, these data reveal that AA specifically sensitizes human ALT cells to ionizing radiation as compared to telomerase-positive cell lines.

## DISCUSSION

We have previously shown that the knockdowns of the two homologous lysine acetyl transferases GCN5 and PCAF had opposite effects on the alternative mechanism of telomere maintenance, suggesting that PCAF, which is absent from telomeres may indirectly favor ALT, whereas GCN5, which is present at telomeres, may oppose to telomere recombination [13]. Here, we report that the pan-inhibitor of lysine acetyl transferases AA recapitulates only the effects of PCAF knockdown on ALT cells. We have shown that PCAF is involved in the formation of APBs, which are not only a marker for ALT cells but play a direct role in telomere recombination, both by bringing together chromosome ends and by promoting telomere-telomere interactions between heterologous chromosomes [23]. These results indicate that PCAF favors ALT through its lysine acetyltransferase activity whereas GCN5 opposes ALT by another mechanism. We hypothesized previously that GCN5 may act through its interaction via the SAGA complex with the deubiquitinase USP22 in APBs to prevent ubiquitin-mediated proteolysis of telomere proteins and therefore to oppose telomere recombination [12, 13].

PCAF forms activating transcriptional complexes with other partners, including p300, which bind promoters and activate transcription by prompting histone acetylation [29, 30]. AA decreased APBs formation similarly to PCAF or P300 knockdowns [13]. Moreover, this treatment decreased mRNA levels of some shelterin proteins required for the APB assembly [26]. Therefore, we speculate that PCAF and P300 are involved in the regulation of the ALT activity through the transcriptional modulation of genes implicated in APB formation. Interactions of PCAF and p300 have also been shown to activate the expression of BRCA1 [31], known to be recruited in APBs and to promote ALT [32–34]. Transcriptional downregulation of BRCA1 expression by the inhibition of the lysine acetyl transferase activity of the PCAF and P300 complex may thus be part of an integral signaling pathway that also determines and explains the downregulation of the ALT phenotype upon AA treatment.

Several studies have shown that AA radiosensitizes various cancer cell lines, cervical adenocarcinoma, squamous carcinoma cell lines and the ALT cell line U2OS (human ALT osteosarcoma) [27, 28]. Among the possible mechanisms, AA has been proposed to repress H2AX expression in prostate cancer cells [16] or to reduce the expression of anti-apoptotic proteins associated with cell survival and radioresistance in pituitary adenoma cells [18]. Here we show that ALT cells are particularly radiosensitized by AA as compared to telomerase-positive cells, suggesting that ALT inhibition enhances the cell sensitivity to radiation. This is consistent with previous reports showing that telomere dysfunction impairs DNA repair and enhances sensitivity to ionizing radiation [35]. In particular, suppression of telomere-binding protein TPP1 (TIN2 and POT1 interacting protein) has been shown to result in telomere dysfunction and to enhance radiation sensitivity in ALT cell lines [36].

Our data suggest that inhibition of lysine acetyl transferases could represent a new therapeutic target against ALT cancer cells. ALT is frequent in Glioblastoma multiforme (GBM) [1, 2], a primary brain tumor with a very poor prognosis. Tumor relapses are attributed to the resistance of GSCs to standard treatments, which include surgical resection, radiotherapy and chemotherapy using Temozolomide (TMZ). We have shown that AA not only impaired the viability of the ALT GSC line TG20, but also markedly increased its radiosensitivity. Interestingly, TG20 has been previously shown to be particularly radiation resistant [19] and resistant to TMZ [37]. Our data suggest therefore that the targeting of the lysine acetyl transferases may represent a promising alternative to the treatment of ALT cancers.

## MATERIALS AND METHODS

### Cell culture and reagent

Telomerase-positive GSCs, including the TG16, TG1N and TG10 cell lines, and the ALT GSC TG20 line were maintained in culture as previously described [19]. The human osteosarcoma ALT cell line SAOS*-*2 was obtained from the American Type Culture Collection (HTB85, ATCC) and was grown in DMEM (Gibco, Life Technologies) supplemented with 10% fetal bovine serum (PAA Laboratories). For AA exposure, the drug was purchased from Cell Signaling and dissolved in dimethyl sulfoxide (DMSO) (maximum concentration: 143 mM) as a stock solution and prepared in dilution with culture when necessary. Effects of AA were systematically compared to DMSO controls.

### WST-1 cell proliferation assay

Cells were plated in 96-well plates for 72 h with the AA treatment. A total of 2,000 cells were used per well. After 72 h of AA treatment, 10 μl of WST-1 (ref 11644807001, Roche) reagent was added to each well and incubation was continued for an additional 2–3 h. The absorbance was determined using a spectrophotometer (Bio-Tek uQuant *MQX200* Microplate Reader Spectrophotometer) at a wavelength of 450 nm.

### Cleaved-caspase 3 detection

Anti-activated Caspase-3 antibody (1:200, Cell Signalling Technology #9661) was used to examine apoptosis induced in different cells. Briefly, cells were grown on Labteck chamber slides (PEZGS0816, Millipore) for 24 hours, and then treated with AA for 72 h. After that, treated and controls cells were fixed with 4% paraformaldehyde and incubated with the antibody and then examined under fluorescence microscope (Olympus U-RFL-T).

### Population doubling assay

Cells were seeded on 6-well plates and passaged every 3 days upon reaching 70% confluence. The number of cells at every passage was counted by the Trypan Blue exclusion method and the AA was added after each passage. The PD was calculated using equation log2 (harvested cells number/ plated cells number). The final PD for each passage was determined by successive addition of total numbers in each passage.

### Western blot

After 72 h of AA treatment cells were lysed by RIPA Lysis Buffer containing protease and phosphatase inhibitor. An equal amount of each protein sample was separated by 8–12% SDS–PAGE and transferred onto PVDF membranes. The following antibodies were incubated with the blocked membrane to detect the proteins of interest: anti-acetyl histone 3 (1:1000, Millipore 06-599), anti-histone 3 (1:2500, ABCAM ab1791), anti-PCAF (1:1000, Santa Cruz 13124) and β-actin (1:1000, Sigma-Aldrich A1978).

### PCAF and GCN5 knockdowns

Selected small interfering RNA (siRNAs) targeting PCAF and GCN5 also negative control duplex siRNAs (siCtrl) (Life Technologies) used in this study were previously described and validated in Jeitany *et al.* 2017. The following siRNA constructs were as follows: siPCAF sense, 5′-CCACUUUAAUGGGAUGUGAGCUAAA-3′ and antisens, 5′-UUUAGCUCACAUCCCAUUAAAGUG G-3′; siGCN5 sense, 5′-CCAAGCAGGUCUAUUUCU ACCUCUU-3′and antisense 5′-AAGAGGUAGAAAUAG ACCUGCUUGG-3′ (Life Technologies). Briefly, cells were dissociated and transfected via electroporation with a Neon Transfection System (Life Technologies) using the manufacturer’s protocol. Transfected cell combined or not with AA treatment were plated 72 hours on laminin-coated flasks (for chromosome orientation-fluorescence *in situ* hybridization (CO-FISH) experiments) or Millicell EZ slides (PEZGS0816, Millipore, for immunofluorescence (IF) assays).

### Stable knockdown of PCAF in SAOS-2 cells

SAOS-2 cells were stably transfected with Epstein-Barr virus (EBV) vectors expressing shRNA targeting PCAF (named here SAOS-2 shPCAF) that were designed by D. Biard as described in a previous study [38, 39]. The following shRNA sequence was: 5′-GCAAATAATTGTCAGTCTA-3′ (position in the ORF: 378-396). Negative control EBV vectors expressing a previously described inactive shRNA sequence were also stably transfected into SAOS-2 cells (SAOS-2 shCTR) [38, 39]. Knockdown efficiencies were determined by quantitative RT-qPCR for RNA expression and by western blot for protein expression as described. SAOS-2 shCTR and SAOS-2 shPCAF were transfected with siGCN5 or siCTR and then plated for 72 hours on Millicell EZ slides (PEZGS0816, Millipore), for APB assays.

### Immunofluorescence and detection of ALT associated PML bodies (APBs)

Cells were treated with AA or DMSO and fixed in 4% paraformaldehyde. Cells were then permeabilized in 0.5% Triton X-100 in phosphate-buffered saline (PBS) and blocked in 7.5% Goat Serum + 7.5% fetal bovine serum. The blocked cells were then incubated with anti-PML (1:100; Santa Cruz Biotechnology sc-966) overnight at 4° C. The next day, cells were washed with PBS and incubated with secondary antibody for 1h at room temperature. The slides were mounted with Dapi Fluoromount-G (Southern Biotech). Images were taken using the Leica DM 2500 microscope.

For the detection of APBs, the immuno-staining of telomeres was performed after the staining of PML protein described above, by fixation in 4% formaldehyde and dehydration with ethanol (50°–80°–100°). The dehydrated slides were overlaid with Cy3-(CCCTAA)3 PNA probe (Applied Biosystems) prepared in PNA hybridization solution (70% formamide - 10 mM Tris pH 7.2, BSA 1%), then incubated at 80° C for 3 min and hybridized at room temperature for 2 hours. The slides were washed twice in 70% formamide-10 mM Tris pH 7.2 and three times with 50 mM Tris pH 7.2–150 mM NaCl-0.05% Tween-20. Finally, the slides were mounted with Dapi Fluoromount-G (Southern Biotech). Images were taken using the Leica DM 2500 microscope.

### CO-FISH

CO-FISH allows the detection of T-SCEs and was performed as previously described [40]. Using this technique, the chromosome telomere lagging strands (TTAGGG) and telomere leading strands (CCCTAA) can be differentiated after selective degradation of the neosynthesized strands and the use of specific fluorescent PNA probes. Briefly, cells were grown in the presence of BrdU for one cell cycle. The cells were spread on SuperFrost Plus glass slides (Menzel-Glaser) and stained with Hoechst 33258. Then, they were exposed to UV light and degraded by exonuclease Exo III (Promega). After dehydratation with ethanol (50°–75°–100°) slides were hybridized (2 hours at room temperature) with a telomeric FITC-conjugated (T2AG3) PNA probe (Applied Biosystems) that is complementary to the C-rich telomeric strand (leading strand). Incubation (2 hours at room temperature) with a telomeric Cyanine-3-conjugated (C3AT2)3 PNA probe (Applied Biosystems) that is complementary to the G-rich telomeric strand (lagging strand) followed. Metaphases were captured and analysed using an Axio Imager Z.2 (Zeiss, Oberkochen, Germany) coupled to a Metafer Image Analysis System (MetaSystems, Altlussheim, Germany).

### Quantitative RT-PCR

Total RNA was extracted using an RNeasy Plus Mini Kit (Qiagen) according to the manufacturer′s instructions and reverse-transcribed to cDNA using High Capacity RNA-to-cDNA Master Mix (Applied Biosystems). The following primers were used:

*pcaf* primers: forward: 5′-GCCACAGTTCT GCGACAGTCT-3′, reverse: 5′-CCGAGCGAAGCAATG TTCTC-3′ (EuroGentec);

*gcn5* primers: forward: 5′-GTGCTGTCACCTC GAATGAG-3′, reverse: 5′-TGGAGAAACCCTGCTTTT TGA-3 (EuroGentec);

*trf1* primers: forward: 5′-CCTTATTGAGGTCTC ACAAGAATC-3′; reverse: 5′-CTGCTTTCAGTGGCTC TTCTGC-3′ (EuroGentec);

*trf2* primers: forward: 5′-GTACCCAAAGGC AAGTGGAA-3′; reverse: TGACCCACTCGCTTTCTT CT-3′ (Sigma);

*rap1* primers: forward: 5′-ATAGCGGGGAACCAC AGAATAAC3′; reverse: 5′-ACCACAACCTCCTCA AACTCCC-3′ (EuroGentec);

*tin2* primers: forward: 5′-ACTAGGGGAGGCCAT AAGGA-3′; reverse: 5′-GGGCTGGCATGGACTCTTA-3′ (Sigma).

The results were normalized to *gapdh* levels, which were determined with the primers: forward:5′-GTCGCCAGCCGAGCCACATC-3′; reverse: 5′-GGTGA CCAGGCGCCCAATACG-3′. The reactions were performed with a SYBR Green Kit (Applied Biosystems).

### Colony formation assay

Clonogenic assay was used to assess the effect of AA in combination with radiation. Cells were plated in 6-well plates. Once they are attached, they were treated with AA for 24 h or 72 h. Cells were then irradiated with 0, 2 or 4 Gy gamma radiation (GSR D1, gamma service) and then cultured for 14 days, at which point colonies could be identified by the naked eye. The cells were subsequently fixed in acetic acid/methanol and stained with crystal violet. Colonies were counted manually or using the Typhoon software and the clone formation efficiency was calculated as the ratio of clone numbers to plated cell numbers in each well.

### Statistical analysis

All statistical tests and graphical drawings were performed on GraphPad PRISM version 7. Results are expressed compared to the vehicle within the same cell line, by the Student’s *t*-tests or by analysis of variance (ANOVA).

## SUPPLEMENTARY MATERIALS FIGURES


