# Supplementary material for: ALT cancer cells are specifically sensitive to lysine acetyl transferase inhibition
Source: Oncotarget. 2019 Jan 22;10(7):773–84. doi: 10.18632/oncotarget.26616 (PMC6366824; doi:10.18632/oncotarget.26616)
Supplement: Supplementary file 1 [file oncotarget-10-773-s001.pdf]

ALT cancer cells are specifically sensitive to lysine acetyl transferase inhibition

SUPPLEMENTARY MATERIALS

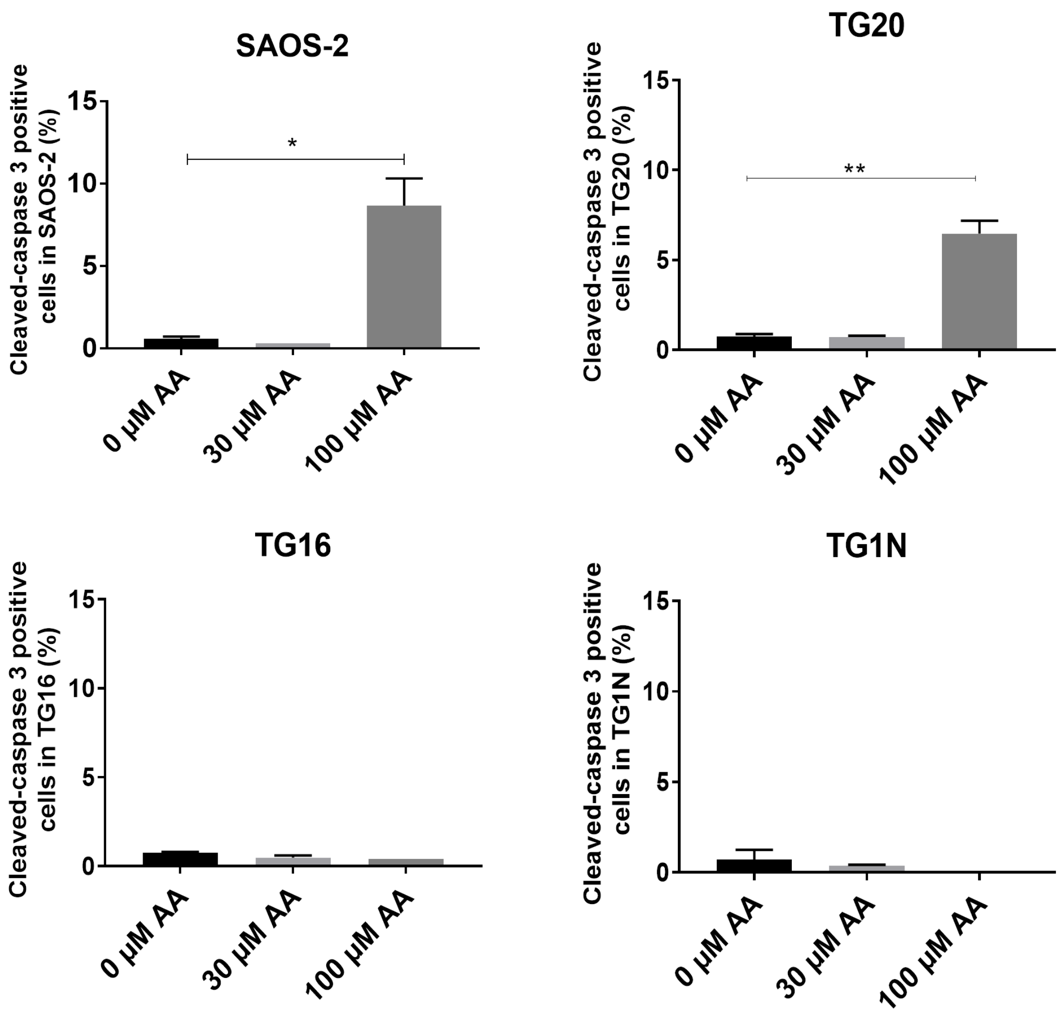

Supplementary Figure 1: Detection of caspase-3 in apoptotic cells after AA treatment. Quantification of the cleaved caspase-3 positive cells in SAOS-2 (upper left), TG20 (upper right), TG16 (lower left) and TG1N (lower right) after treatment with 30 µM or 100 µM of AA for 72 hours relative to untreated control cells. Columns represent the average of two different experiments, each done at least in duplicate and for each condition at least 150 cells were analyzed. ($^{*}p < 0.05$, $^{**}p < 0.01$ referred to *t*-test).

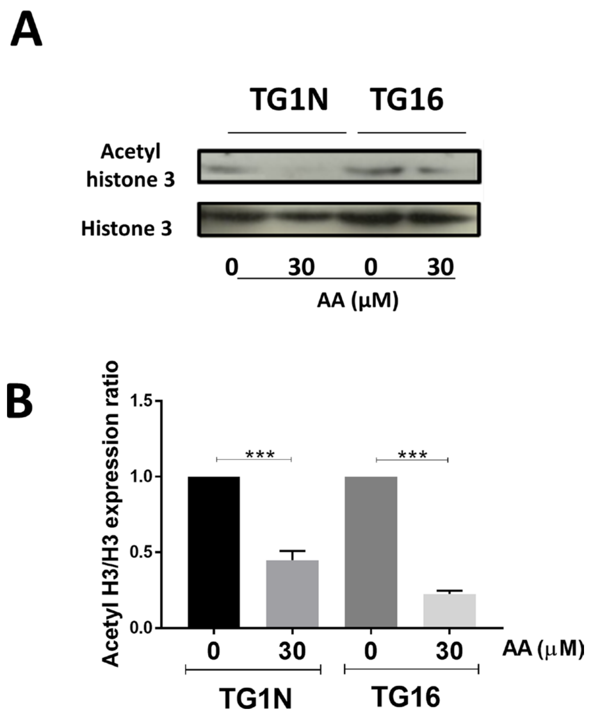

Supplementary Figure 2: Effect of AA on acetylated histone 3 in telomerase positive cancer cells. (**A**) Western blot analysis of acetylated histone H3 performed in telomerase positive (TG1N and TG16). Cell extracts 72 h after 30 μM AA treatment. The untreated controls contained 0.1% DMSO. (**B**) The quantitative data are shown as relative intensity of acetylated histone band in arbitrary units that was adjusted for total histone 3 intensity and normalized to those of the control untreated. Data are expressed as the means ± SD of a least two independent experiments for each cell line. ($^{***}P < 0.001$ compared with the untreated cells as determined by Tukey-Kramer one way Anova).

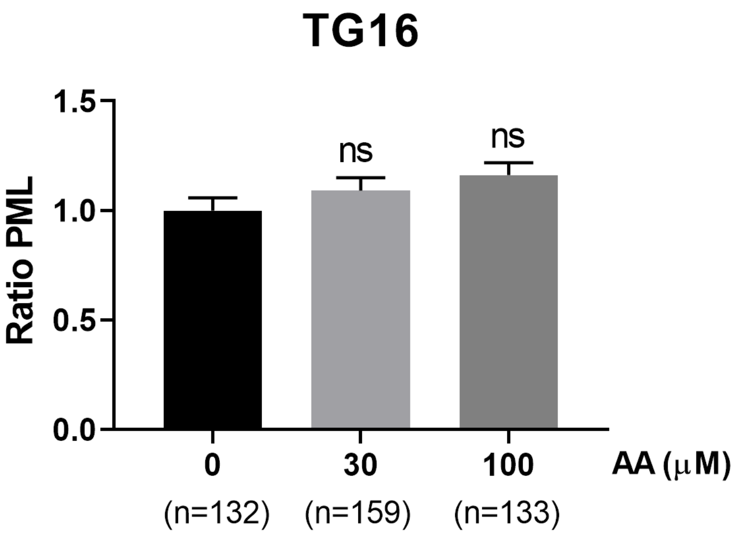

Supplementary Figure 3: Effect of AA on the number of PML bodies in telomerase positive human glioma cells. PML bodies were scored in TG16 telomerase positive cancer cells treated or not with AA. "*n*" indicates the number of counted cells. The values represent the ratio of number of PML bodies per cell (+SEM) relative to untreated for each cell line. (ns $p > 0.5$ as determined by Student's *t*-test).

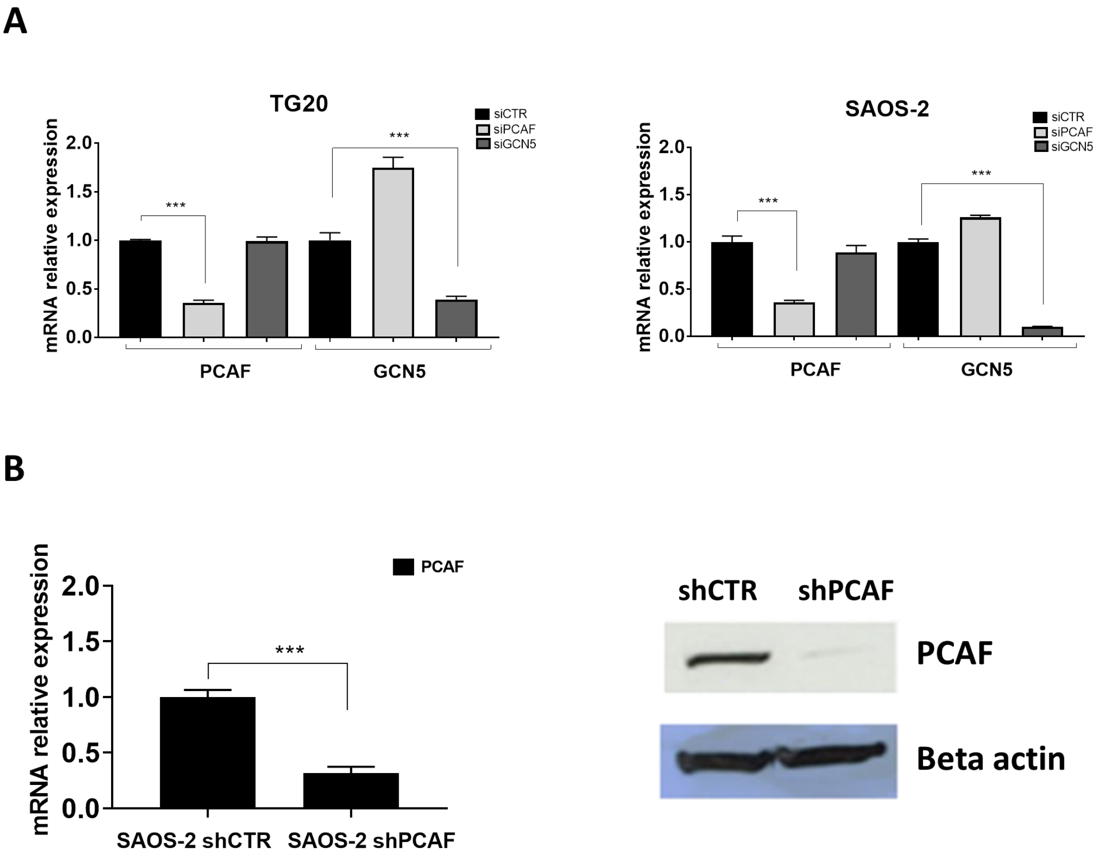

Supplementary Figure 4: Expression profiles of PCAF and GCN5 in used cell lines. (**A**) mRNA expression levels of PCAF and GCN5, as determined by qRT-PCR, in TG20 (left) and SAOS-2 (right) cells transfected 72 h with siPCAF or siGCN5 relative to their expression in cells transfected with siCtrl, proving the efficiency and specificity of the siRNAs. The error bars are the SEM from three experiments. (**B**) PCAF protein expression *via* Western blot analysis (right) and mRNA expression via RT-PCR analysis (left) in SAOS-2 cell lines transfected with shCTR or shPCAF.
